# Biological networks: the microscope of the twenty-first century?

**DOI:** 10.3389/fgene.2015.00307

**Published:** 2015-10-13

**Authors:** Frank Emmert-Streib, Matthias Dehmer

**Affiliations:** ^1^Computational Medicine and Statistical Learning Laboratory, Department of Signal Processing, Tampere University of TechnologyTampere, Finland; ^2^Institute of Biosciences and Medical TechnologyTampere, Finland; ^3^Department of Computer Science, Universität der Bundeswehr MünchenGermany; ^4^Department of Mechatronics and Biomedical Computer Science, UMITHall in Tyrol, Austria

**Keywords:** biological networks, computational biology, network biology, translational research, systems medicine

The development of science is accompanied by a number of seminal inventions that contributed significantly to make science what it is today. In biology and the biomedical sciences, the introduction of the light microscope in the seventeenth century by Antony van Leeuwenhoek and others can certainly seen as such a contribution because it literally opened a door to a new universe that cannot be seen by the naked eye, the molecular world of biology (Wollman et al., 2015). Within 100 years after its introduction, new phenomena in biology and medicine have been discovered, e.g., different types of cells such as red blood cells or the existence of single-cell organisms like bacteria. Furthermore, performing tissue analysis enabled histopathology and new concepts of diseases based on cells rather than whole organs.

Extending the principle idea of the microscope, a variety of imaging technologies have been introduced since then allowing to visualize for instance, the 3D-structure of a protein (crystallography), broken bones (x-ray), the structure of atoms (electron microscope), or the neuronal activity of the brain (MRI). Despite the technical differences between all of these different imaging technologies they share the common principle idea to enhance the capabilities of our eyes by making them more sensitive to the scale of the microscopic and atomic world. Hence, this allows it for everyone to easily grasp the capabilities of any of the above imaging methods and to understand the resulting visualizations as a direct reflection of reality.

A recent publication by Cohen (2004) attracted wide attention, because the author put the provocative argument forward that *mathematics is biology's next microscope*. In our paper, we provide a succinct example for this. In our opinion *biological networks* are a prime example for a mathematical approach that has indeed much in common with a microscope, but differs in one important point making them even more potent for biology and medicine. Namely, biological networks are mathematical models, as we will argue below.

The history of networks, or graphs in general, started at about the same time as that of the microscope with Euler studying the Königsberger bridges problem (Seven Bridges of Königsberg; Euler, 1736) while the term “graph” was coined by König much later in the 1930s (König, 1936). The first wave of general interest outside mathematics was sparked by the introduction of *random networks* in the 1950s (Solomonoff and Rapoport, 1951; Erdös and Rényi, 1959) followed by the second wave in the mid 1990s discovering that many different types of “real” networks have structural properties quite different from random, which can be better described by scale-free and small-world networks (Watts and Strogatz, 1998; Barabási and Albert, 1999). One highlight for the application of random networks in biology can be found in the seminal work of Kauffman (1969) introducing models for gene regulations, whereas scale-free and small-world networks helped shaping the emergence of systems biology, network biology, and network medicine (Barabási et al., 2011). Fueled by big data from genome-scale screening experiments in combination with powerful statistical inference methods this enabled finally the emergence of gene regulatory networks for mammalian organisms including Human (Basso et al., 2005; Emmert-Streib et al., 2014). Note that graph-theoretical methods such as graph comparison to analyze biological networks have been discussed in Emmert-Streib and Dehmer (2011).

A similarity between microscopes and networks is that both allow the visualization of an underlying phenomenon so it can be explored by means of the eyes. However, a fundamental difference is that microscopes visualize *geometric objects* whereas networks show *topological objects*, see Figure 1. The difference is that neither the nodes/vertices of a graph nor their edges/links are associated with 2- or 3-dimensional coordinates that specify their Euclidean position, e.g., on a canvas or screen uniquely, but their positions are not defined by the definition of the graph at all. In other words, a graph is arbitrarily deformable with respect to the positions of its constituting components.

**Figure 1:**
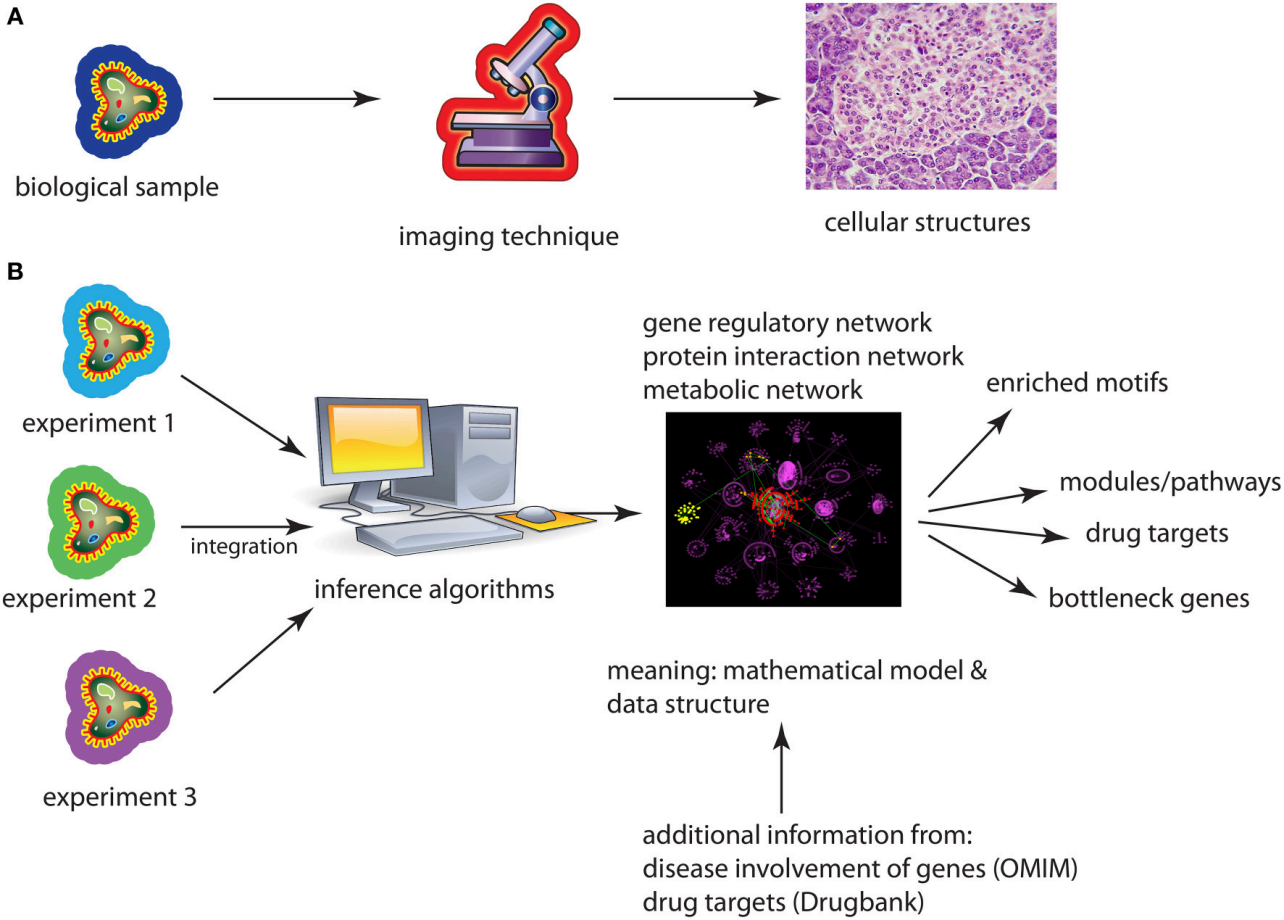
**(A)** Application of an imaging technique results in a re-scaled image of the underlying biological sample. **(B)** Application of an inference method results in a biological network either from one or several different data sets. A subsequent analysis of the network provides then biological information about the used data sets coming from cell lines, model organisms, or patients.

The implications that result from this fundamental difference are enormous and we want to highlight just three examples. First, networks are not merely a magnification of a microscopic world in a different scale, like a model car. Instead, networks are mathematical models that cannot be observed with a microscope at all, even with an hypothetically large magnification. For instance, a transcriptional regulatory network (TRN) is a directed network that represents the regulations of transcription factors on the transcription of genes on a genomic-scale. While it is true that one specific transcription factor binds at a particular time and space to the promotor region of a gene to initiate its transcription, not all transcription regulation events happen at this particular time or space. Also, no matter how long you observe such events, they never happen all at once. As such the TRN does not represent what can be observed but it corresponds to averaged observations over time and space making it a mathematical model of transcription regulation. Second, since all types of biological networks are *models*, which are per see abstract, one needs actually to derive the information captured in them by application of abstract (mathematical/statistical) methods. Examples for such an information extraction analysis step are the identification of modules/pathways, identification of bottleneck genes or the detection of evolutionary conserved motifs (Milo et al., 2002; Yu et al., 2007). Third, considering networks as data structures (Emmert-Streib and Dehmer, 2011) allows a convenient integration of high-throughput data from different experiments, e.g., by the application of Bayesian methods. This also allows the integration across heterogeneous data types, e.g., from gene expression and proteomics, resulting in hierarchical, multi-scale models of molecular interactions.

We think that the long standing visualization tradition in biology and medicine by means of microscopes causes also one of the biggest misconceptions of biological networks. Namely, that their purpose is their visualization to “see” what information the networks contain. However, the fact that one can visualize biological networks does not establish this as their main purpose. Instead, the main purpose of any mathematical model is to be used for extracting information from it, and a visualization is just one side of this, e.g., by enabling an exploratory analysis (Tukey, 1977).

We are of the opinion that, so far, biological networks are not utilized up to their full potential and there are at least three reasons for this. First, when biological networks are inferred from data it is not clear where to deposit them so they can be re-used by other groups. In contrast, when generating gene expression or next-generation sequencing data there are a number of well known databases that are open to anyone to deposit the data, e.g., GEO or Ensembl. These databases enforce also the documentation of the data in a standardized way that ensures an easy re-use of the data with a minimal (ideally no) interaction with the data generating groups. At the moment, it is unclear where to store inferred biological networks, in what format and what additional information needs to be provided because, presently, there are no official standards. However, the R packages igraph and graph (Csardi and Nepusz, 2006; Gentleman et al., 2010) should be notably mentioned for providing low level graph operations. In addition, depositing networks into databases would be also an important step toward reproducible results in this area.

Second, due to the lack of databases from which biological networks can be acquired, necessarily, an analysis starts with the inference of the networks. Unfortunately, the application of causal inference algorithms to experimental or observational data (Emmert-Streib et al., 2012) is far from being trivial leading frequently to low quality networks, even when starting with high quality data. This makes the resulting downstream analysis at least problematic or even controversial. Due to the intricacy of this analysis step, we suggest a collaborative approach by teaming up with a computational biology or biostatistics group that is specialized in such an analysis in order to ensure the highest quality of the inferred networks and all derived subsequent results.

As a third issue, we think that complementing biological networks with additional information, e.g., from associations between genes and disorders (OMIM) or drug targets (Drugbank) would be very beneficial for studying problems beyond biology, e.g., in translations medicine (Dudley and Karczewski, 2013). Notable example studies can be found in Ideker and Sharan (2008); Kotlyar et al. (2012). This connection would allow to bring a systems approach down to the patient side, e.g., for investigating diagnostic or prognostic questions. Furthermore, in this way, naturally, a door to pharmacogenomics is opened placing the drug targets into their molecular interaction networks. In this way, new insights into the side effects of drugs may be gained.

Returning back to our initial analogy, biological network research is surely still at the stage of the early microscope and it maybe too early to state without uncertainty that biological networks will indeed acquire the status of a microscope. However, implementing some of the above measures will quickly allow biological networks to further blossom in biological, medical, and pharmacogenomic research to drive these fields to a level unachievable without the usage of biological networks.

## Author contributions

FE and MD conceived the study, wrote the paper, and approved the final version.

## Funding

MD thanks the Austrian Science Funds for supporting this work (project P26142). MD gratefully acknowledges financial support from the German Federal Ministry of Education and Research (BMBF) (project RiKoV, Grant No. 13N12304).

### Conflict of interest statement

The authors declare that the research was conducted in the absence of any commercial or financial relationships that could be construed as a potential conflict of interest.
